# Supplementary material for: The interactome of intact mitochondria by cross-linking mass spectrometry provides evidence for coexisting respiratory supercomplexes
Source: Mol Cell Proteomics. 2017 Dec 8;17(2):216–32. doi: 10.1074/mcp.RA117.000470 (PMC5795388; doi:10.1074/mcp.RA117.000470)
Supplement: Supplemental Data [file supp_RA117.000470_133922_0_supp_23978_fzffwf.zip › spectra_annotation/mito_DR_spectra_annotation/187-1-8-1-3-1.pdf]

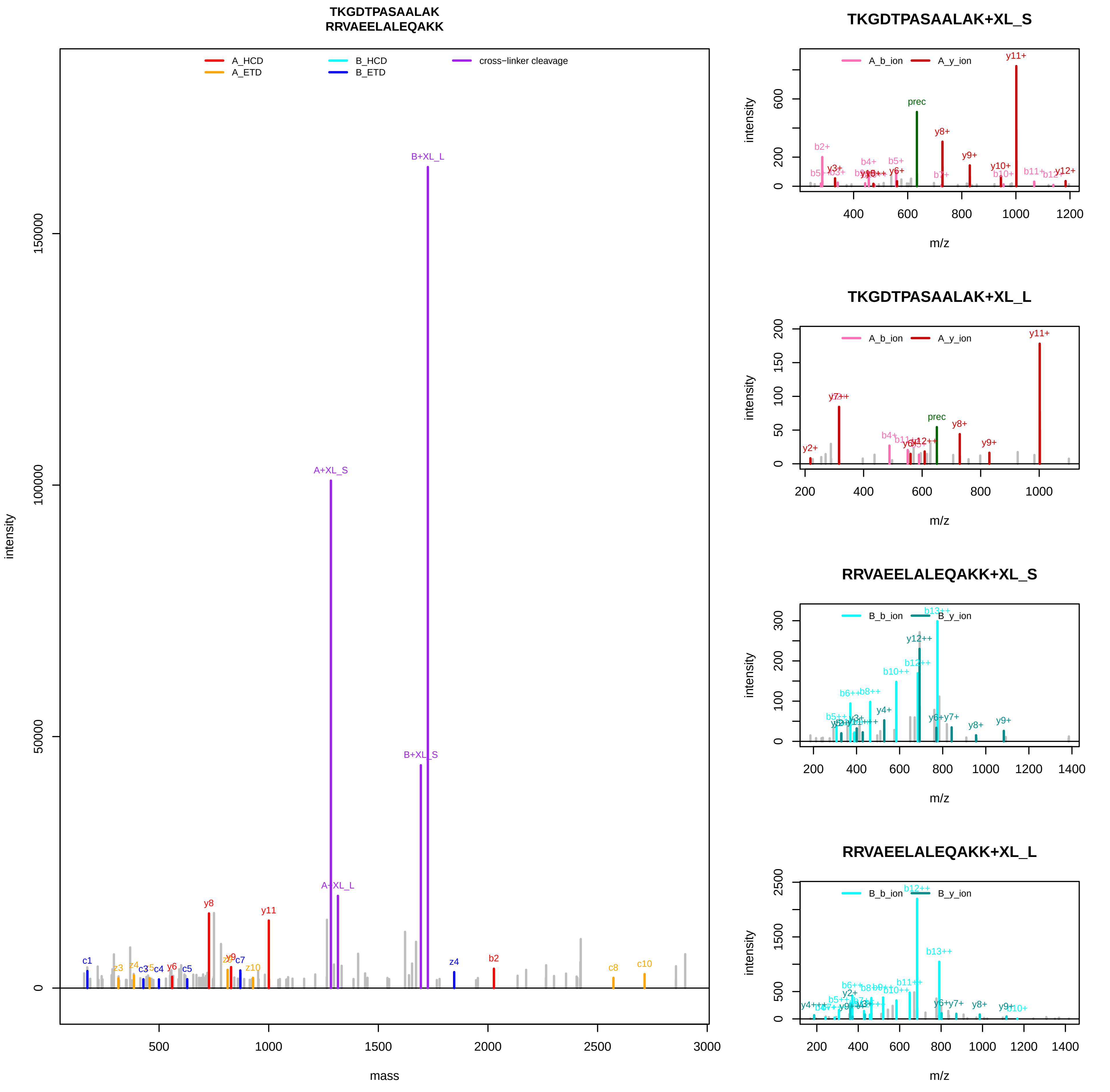
TKGDTPASAALAK
RRVAEELALEQAKK
A_HCD
A_ETD
B_HCD
B_ETD
cross−linker cleavage
B+XL_L
A+XL_S
B+XL_S
A+XL_L
intensity
mass
0
50000
100000
150000
500
1000
1500
2000
2500
3000
c1
z3
z4
c3
c4
c5
y6
y8
y9
c7
z10
y11
z4
b2
c8
c10
TKGDTPASAALAK+XL_S
A_b_ion
A_y_ion
prec
y11+
y8+
y9+
y10+
b2+
b4+
b5+
y6+
y3+
b7+
b11+
b12+
y12+
intensity
m/z
TKGDTPASAALAK+XL_L
y11+
y7+
prec
y8+
y9+
y2+
b4+
intensity
m/z
RRVAEELALEQAKK+XL_S
B_b_ion
B_y_ion
b13++
y12++
b12++
b10++
b6++
b8++
b5++
y4+
y3+
y6+
y7+
y8+
y9+
intensity
m/z
RRVAEELALEQAKK+XL_L
B_b_ion
B_y_ion
b12++
b13++
b6++
b11++
b10++
y2+
y6+
y7+
y8+
y9+
b10+
intensity
m/z
